# Extracellular vesicles and microvilli in the immune synapse

**DOI:** 10.3389/fimmu.2023.1324557

**Published:** 2024-01-03

**Authors:** Javier Ruiz-Navarro, Víctor Calvo, Manuel Izquierdo

**Affiliations:** ^1^ Department of Metabolism and Cell Signaling, Instituto de Investigaciones Biomédicas Sols-Morreale (IIBM), Consejo Superior de Investigaciones Científicas (CSIC)-Universidad Autónoma de Madrid (UAM), Madrid, Spain; ^2^ Departamento de Bioquímica, Facultad de Medicina, Universidad Autónoma de Madrid (UAM), Madrid, Spain

**Keywords:** T lymphocytes, immune synapse, extracellular vesicles, actin cytoskeleton, microvilli, FMNL1β, protein kinase C δ, multivesicular bodies

## Abstract

T cell receptor (TCR) binding to cognate antigen on the plasma membrane of an antigen-presenting cell (APC) triggers the immune synapse (IS) formation. The IS constitutes a dedicated contact region between different cells that comprises a signaling platform where several cues evoked by TCR and accessory molecules are integrated, ultimately leading to an effective TCR signal transmission that guarantees intercellular message communication. This eventually leads to T lymphocyte activation and the efficient execution of different T lymphocyte effector tasks, including cytotoxicity and subsequent target cell death. Recent evidence demonstrates that the transmission of information between immune cells forming synapses is produced, to a significant extent, by the generation and secretion of distinct extracellular vesicles (EV) from both the effector T lymphocyte and the APC. These EV carry biologically active molecules that transfer cues among immune cells leading to a broad range of biological responses in the recipient cells. Included among these bioactive molecules are regulatory miRNAs, pro-apoptotic molecules implicated in target cell apoptosis, or molecules triggering cell activation. In this study we deal with the different EV classes detected at the IS, placing emphasis on the most recent findings on microvilli/lamellipodium-produced EV. The signals leading to polarized secretion of EV at the synaptic cleft will be discussed, showing that the IS architecture fulfills a fundamental task during this route.

## Introduction

### Antigen recognition and immune synapse formation by T lymphocytes

**Figure 1:**
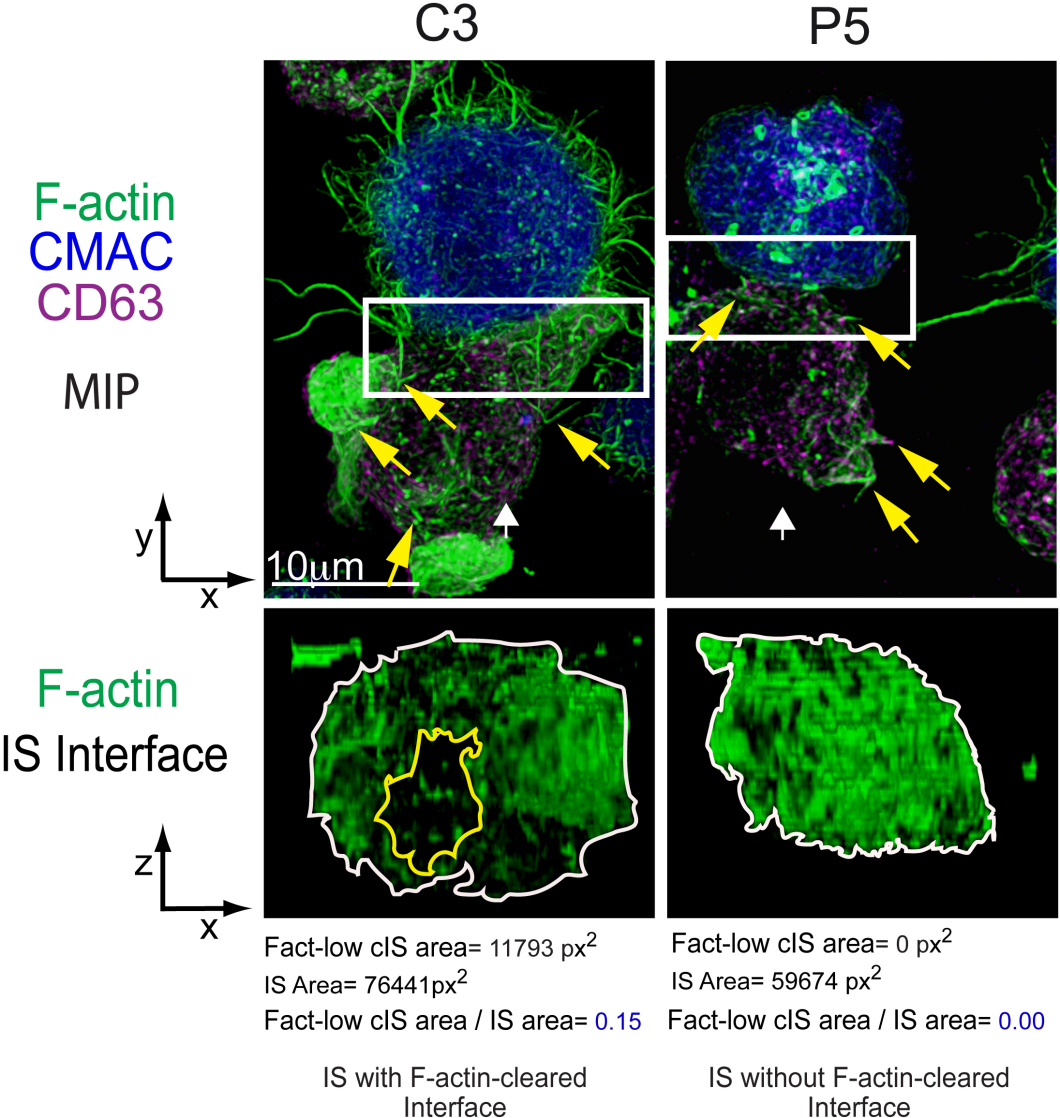
Immune synapse, microvilli and F-actin depletion at the cIS. C3 (control) and P5 (PKCδ-interfered) Jurkat clones were challenged with Cell tracker blue (CMAC)-labelled Raji cells (blue) pulsed with *Staphylococcal* enterotoxin (SEE) to induce IS formation. After 1 h of conjugate formation, fixed cells were labelled with phalloidin (green) and anti-CD63 (magenta) to label F-actin and MVB, respectively. Upper panels: Maximal Intensity Projections (MIP) of merged CMAC, anti-CD63 and phalloidin channels. Lower panels: enlarged XZ plane area of the IS interface area (1.5x and 2.5x zoom for C3 and P5, respectively, from the white rectangles shown in the upper planes) of the phalloidin channel, generated as shown in **Supplementary Video 2**. White arrows indicate the direction to visualize the IS interface views enclosed by the regions of interest (ROIs, white rectangles). Yellow arrows label some microvilli emanating from Jurkat T lymphocytes. The area of the F-actin-low region at the cIS (Fact-low cIS area) (yellow line) and the IS (IS area) (white line) were defined and measured as indicated in (4), and the relative area of the F-actin-low region at cIS (Fact-low cIS area/IS area) was calculated and represented. As seen in the figure, PKCδ is involved in the clearing of F-actin at the cIS, since its depletion impedes the creation of a cortical actin hypodense region at the cIS, which facilitates MVB polarization and secretion (4). This figure is related to **Supplementary Video 2**.

The regulation and execution of adaptive immunity significantly rely on the pivotal involvement of T lymphocytes. To develop their function, T lymphocytes must transiently interact with antigen-presenting cells (APC), searching and sensing through their TCR for cognate antigens presented by major histocompatibility complex (MHC) and through accessory, adhesion molecules including lymphocyte function-associated antigen-1 (LFA-1) interacting with intercellular adhesion molecule 1 (ICAM-1) on APC (1) or CD2 interacting with CD58 (2). This scanning for cognate antigen is carried out by T lymphocytes palpating all points on a stimulatory artificial surface resembling an APC or an APC within roughly one minute through fast movements of their microvilli (3) (**Figure 1**; **Supplementary Video 1**). The time it takes a T lymphocyte to explore the artificial APC’s surface for cognate antigen surveying matches the movement speed of T lymphocytes across tissues (3). These transient interactions occur without TCR recognition, which fulfills the basic trial-and-error attribute of antigen identification, and is stabilized autonomously by integrin signaling or the actin and tubulin cytoskeleton. However, cell-to-cell contact stabilization depends on ligand affinity.

This process, which usually takes 1-5 minutes, enables the T lymphocyte to maintain both close and labile, contact with APC, facilitating the exploration of its plasma membrane for cognate antigenic peptide-MHC (pMHC) complexes (5, 6). When the APC does not bear a cognate antigen the T lymphocyte, owing to its transient and exploratory interactions, detaches from the APC and can explore other APCs presenting cognate antigen (6, 7). This trial-and-error attribute is considered to be a crucial strategy to guarantee the specific binding of the TCR with cognate antigen-presenting APCs (1, 6) and to discriminate the antigen. However, when TCR encounters MHC-presented cognate antigen on APC’s surface, a productive TCR triggering by the antigen presented on APC occurs, causing the establishment of the immune synapse (IS) (8, 9) (**Supplementary Video 1**; **Figure 1**).

### Immune synapse architecture

**Figure 2:**
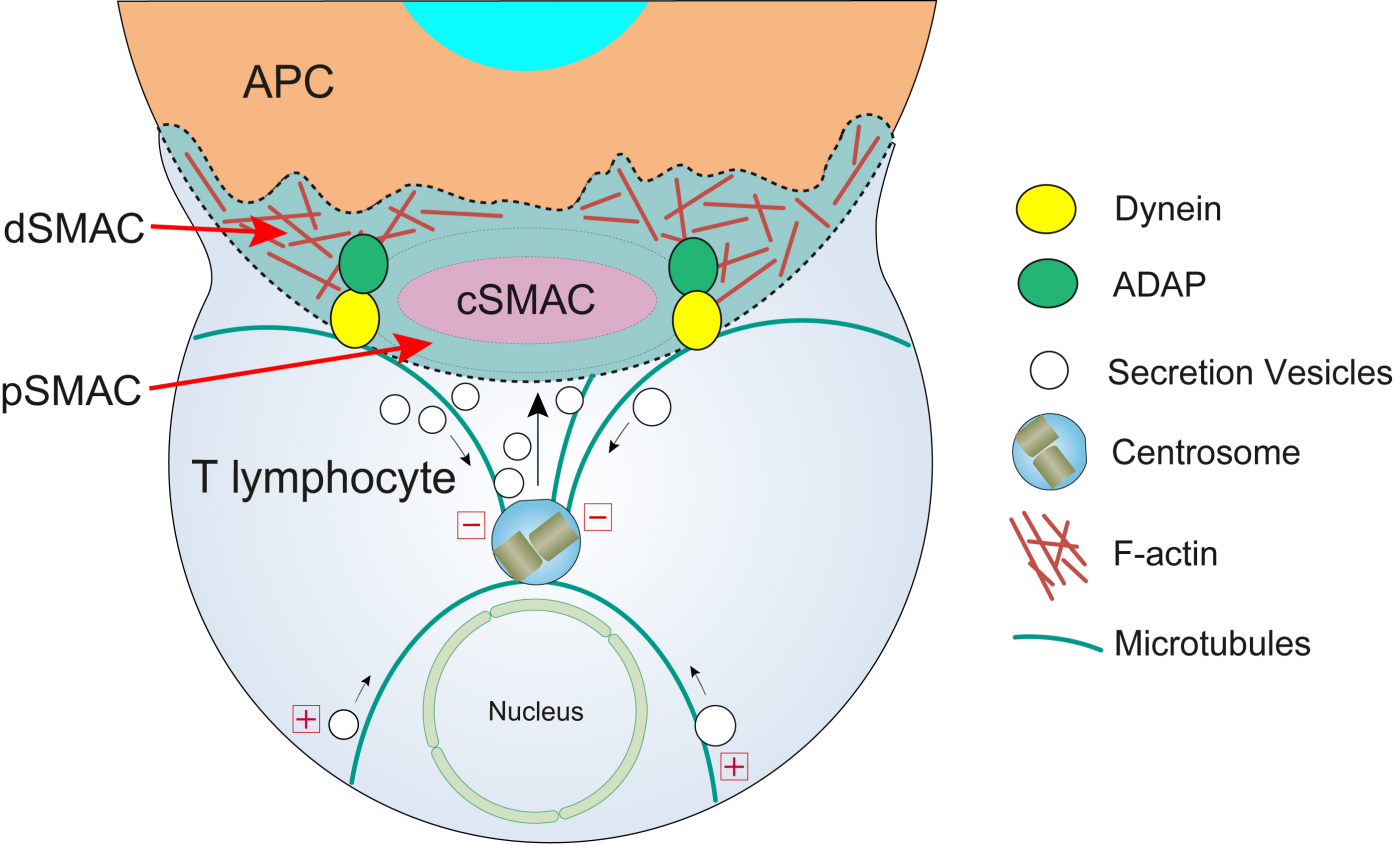
T lymphocyte cortical actin cytoskeleton reorganization and centrosome polarization. After the initial scanning contact of TCR with pMHC on the APC (**Supplementary Video 1**), both naive and effector T lymphocytes form mature IS with APC. Th IS lasts many hours during which *de novo* cytokine (i.e. IL-2, IFN-γ) production and secretion occur, that require continuous TCR signaling. Primed effector CTL establish more transient, mature IS after scanning the target cells (i.e. a virus-infected cell), and secrete cyto lytic granules (CG) within a few minutes. Secretory vesicles (CG in CTL, cytokine-containing vesicles in Th cells, and MVB in both T lymphocyte classes) are rapidly transported (several minutes for effector Th cells and very few minutes or seconds for effector CTL) towards the centrosome (in the minus “-” direction) and, almost simultaneously, the centrosome polarizes towards the cSMAC of the IS, a F-actin poor area that constitutes the secretory domain at the cIS (13). Centrosome translocation to the IS appears to be dependent on DAG production at the IS (14) and DAG-activated PKCθ controlling dynein anchored to adhesion and degranulation adapter protein (ADAP) (15–17) at the pSMAC, that pulls centrosome in the minus direction. In addition, it has been shown that DAG-activated PKCδ controls FMNL1β phosphorylation (18), F-actin clearing at cSMAC and subsequent MVB and centrosome polarization (4). In synapses made by both effector CTL and Th, initial F-actin positive reorganization in the cell-to-cell contact area, and later F-actin decrease at the cSMAC (19) and F-actin accumulation at the dSMAC (20, 21) appear to be involved in centrosome and vesicle polarization and secretion.

IS formation comprises a crucial feature of the immunological system (9). The IS constitutes an accurately and greatly organized, structured, and malleable signaling platform that coordinates cues triggered by TCR along with accessory molecules. This ensures an efficient TCR signal transduction, ultimately facilitating intercellular message transmission. Thus, the IS integrates molecular and cellular interactions, enabling an appropriate antigen-specific response (9). The canonical mature IS developed by both CD4+ T helper (Th) and CD8+ CTL effector cells is characterized by the establishment of a concentric, bullseye-structured, spatial pattern, referred to as supramolecular activation complex (SMAC) (7, 10–12) composed of three compartments (**Figure 2**). First, the central SMAC (cSMAC) where the TCR bound to MHC-presented antigen in microclusters, which were initially formed at the distal SMAC (dSMAC) (see below), are removed, together with signaling molecules, such as the linker for activation of T cells (LAT) and lymphocyte-specific protein tyrosine kinase (Lck). Second, an adhesion molecule-rich region surrounding the cSMAC, where the adhesion molecule LFA-1 is located, termed the peripheral SMAC (pSMAC) (8, 22). Third, the dSMAC, which comprises a dense ring of filamentous actin (F-actin) (13, 23), at the edge of the interaction region with the APC. It is thought that TCR-pMHC microclusters form at the dSMAC and, subsequently, move centripetally though the pSMAC to the cSMAC, initially via F-actin filaments undergoing retrograde flow (23, 24). Finally, the cSMAC acts as a sink of consumed, signaling-incompetent TCR complexes in the process of downregulation (24, 25). Some relevant signaling molecules such as CD45, LAT, and Lck undergo rapid relocalization upon IS formation and these events play a crucial role in modulating TCR signaling. For instance, CD45 phosphoprotein tyrosine phosphatase exclusion from the cSMAC towards the dSMAC plays a crucial role in the initiation of TCR-evoked signaling events (25, 26). This process shifts the equilibrium to the phosphorylated status of the immunoreceptor tyrosine-based activation motifs (ITAMs) located in the TCR/CD3 complex, thereby granting an efficient T cell stimulation (27).

Regarding actin cytoskeleton, the IS establishment is a plastic and dynamic process producing dramatic changes in its organization (28). Initially, a rise in cortical F-actin at the IS region is produced (29) and then, as the synapse developed by effector CTL matures, a reduction in F-actin occurs in the central area of the IS (cIS) which contains the cSMAC, allowing secretion (21). Finally, the secretion of cytolytic granules (CG) at the CTL IS ceases with the F-actin recovery at the cIS (30). Abundant and recent reviews have already dealt with actin cytoskeleton architecture remodeling and its role in lymphocyte polarization (12, 28, 31–36), please consult these reviews for further information.

### Different T lymphocyte immune synapses: some similarities and differences

There is vast diversity among cells that form synapses; therefore, despite the existence of common structural features, the study of the properties of the synapses they form reveals differences (37, 38). The structure of the IS varies with the T lymphocyte differentiation state, type of T cell (CD4^+^ or CD8^+^), primary or immortalized status, type of APC, and the stage of the IS formation (6, 39, 38). Initial T lymphocyte activation at the IS requires priming of naïve T lymphocytes in the secondary lymphoid organs, generally with dendritic cells (DC) acting as APC, which induces metabolic shifts, transcription, translation, proliferation, and differentiation of T lymphocytes into effector and memory subsets, and maturation by acquisition of Th (for CD4^+^ cells) and cytolytic (for CD8^+^ cells) effector functions (32). Subsequently, for initially activated T lymphocytes, serial encounters with pMHC on APC supports cytokine release from the APC and differentiation and maturation of these primed CD4^+^ or CD8^+^ to fully-competent effector T lymphocytes that subsequently will exert their function via IS formation with their target cells (6). Despite its crucial physiological importance on naïve T lymphocyte activation, the IS formed between T lymphocytes and DC has been little studied in comparison with the IS developed by T effector lymphocytes.

Jurkat T CD4^+^ cell line forming IS with *Staphylococcal* enterotoxin E (SEE) superantigen-pulsed Raji cells (**Figure 1**; **Supplementary Video 1**) constitutes an IS model widely accepted by the scientific community as a canonical cell-to-cell synapse Th model that properly reflects a wide number of untransformed human T cells responses (37, 40–43). However, it has been shown that there are differences in the IS actin architecture between Jurkat and primary CD4^+^ T lymphocytes when stimulated in artificial, stimulatory surfaces used as IS surrogates (see below) (38, 44). Whereas the canonical bullseye, monofocal pattern was observed both in Jurkat/SEE-Raji synapse (**Figure 1**) and a primary CD4^+^ clone forming IS with an APC (22), a variation was observed when naïve CD4^+^ T lymphocytes were activated using DC, resulting in a multifocal synapse, illustrating the dependence of the APC on the synaptic actin architecture (45). Although other authors have found common features in the IS architecture between Jurkat and primary, effector CD8^+^ T lymphocytes (42), all these findings support that care should be taken when interpreting or contrasting IS architecture results obtained from different cell types, diverse maturation stage or using different IS surrogates.

Despite all these facts, synapses formed by both effector Th lymphocytes and CTL share important characteristics, such as the reorganization of actin into the canonical bullseye structure, but they have fundamental differences (28). It is out of the range of this study to focus deeply on this subject, therefore refer to superb reviews focused on that topic (9, 2, 11, 38). However, it is remarkable these variations are mainly found in terms of stability and duration, which justify their biological function (46). Previously activated, effector CD4^+^ Th lymphocytes typically develop lasting IS (from minutes up to hours) (5), while effector CD8^+^ CTL form transient IS that leads to the elimination of the target cell and last very few minutes (13, 47). This distinctive property could optimize the function of each cell type; in the IS formed by effector Th lymphocytes, it allows continuous and sustained secretion of stimulating cytokines, while in effector CTL, it enhances the rapid polarization of the secretory organelles and the effective target cells elimination through the secretion of CG or secretory lysosomes (46, 48).

### Microvilli, immune synapse, and antigen recognition

**Figure 3:**
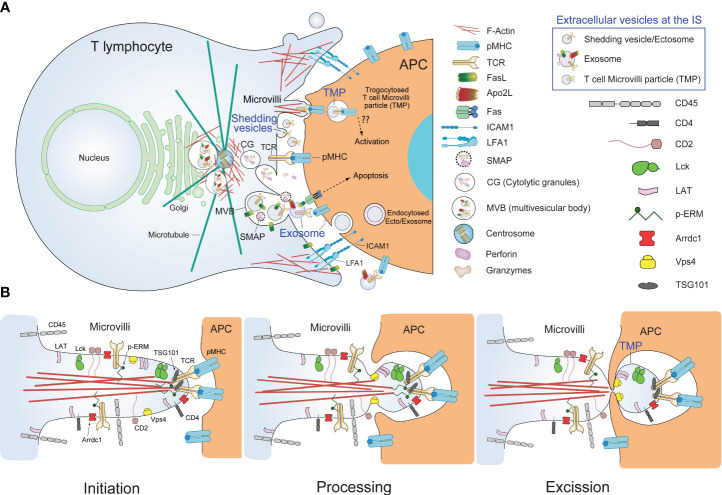
Immune synapse, microvilli and EV. **(A)** Schematic picture illustrating the secretion of different EV, SMAPs and cytotoxic molecules by an effector CTL at the synaptic cleft. A mature IS formed by a CTL and an APC is represented. The different EV (blue characters) and some relevant molecules that can be found at the synaptic cleft are depicted. In addition, canonical CG or single core granules involved in the secretion of soluble perforin and granzyme B at the IS (11, 55, 56) are represented. Depletion of F-actin at the cIS and accumulation at the dSMAC occur, that are concomitant to MVB/centrosome polarization towards the IS and subsequent MVB and CG degranulation, which ultimately leads to exosome (4, 18, 57, 58), perforin/granzyme (11) and SMAP (56, 59) focused secretion. More recently, a new role of centrosomal F-actin on centrosome polarization has been established both in Jurkat cells and primary, effector CD4^+^ T lymphocytes forming synapses with superantigen-loaded Raji cells (18, 36). In addition, ectocytosis of shedding vesicles (ectosomes), via TSG101 and Vps4 (60, 61), and generation of TMPs from microvilli may occur (62, 63). (??) symbol means that is unclear whether TCR on endocytosed TMP may trigger APC activation via pMHC stimulation (62). All these secretion events are represented together to save space in the figure, although is not clear whether these events may simultaneously occur at the same IS. **(B)** Schematic representation of the trogocytosis mechanism and TMP production carried out by an APC acting upon a T lymphocyte. The figure represents three sequential stages of TMP formation via trogocytosis at the IS, namely initiation, processing and scission, as well as the distribution of some relevant T lymphocyte signaling molecules along microvilli (3, 62, 64). TCR, and proteins involved in early T lymphocyte signaling (i.e. Lck, LAT), accumulate in microvilli tips (53, 64, 65) prior to IS formation (66) whereas phosphoprotein phosphatase CD45 is excluded from microvilli tips (27). Molecules relevant for trogocytic TMP scission (Vps4, Arrdc1) remain accumulated into TMPs (64).

The T lymphocyte membrane exhibits an uneven and spatially heterogeneous structure which comprises distinct morphological motifs (49). The main morphological characteristic of the T lymphocyte membrane is the presence of tiny, finger-like cylindrical protrusions named microvilli (3, 49, 50), actin-rich protrusions (51, 52), or actin-rich lamellipodial projections (53, 54). Average microvilli length is 0.3 µm (0.2-5 µm), and their width is between 0.2-0.5 µm (49, 50). These structures cover the surfaces of many cells and are abundant on circulating T and B lymphocyte surfaces (**Figure 1**; **Supplementary Video 1**; **Figure 3**). In fact, microvilli have been found in both effector CD4^+^ and CD8^+^ T lymphocytes (3, 53, 62), but also in naïve T lymphocytes (50). T lymphocyte microvilli are highly dynamic, F-actin-supported protrusions that do not seem to depend on Wiskott-Aldrich syndrome protein (WASp) for their structure (50), although this is not clear for other authors (51, 67).

Regarding their function, it has been established that microvilli enhance the effective surface of T lymphocytes by around 50%, while having very little effect on cytosolic volume, thus favoring APC’s surface scanning and cognate pMHC detection (3, 52). In addition, the efficient survey of APC surface by T lymphocytes conferred by such dynamic protrusions is crucial for establishing a defensive adaptive immunity, and T lymphocyte microvilli are capable of efficiently detecting and discriminating a single cognate pMHC among a myriad of very structurally analogous yet non-cognate, pMHC on the plasma membrane of APCs or target cells (52). Furthermore, super-resolution optical microscopy has revealed that TCR molecules are highly concentrated at the tips of microvilli even in quiescent T lymphocytes, before antigen recognition (65). One study about the ezrin, radixin, and moesin (ERM) protein family has revealed that these proteins co-localize with both TCR and F-actin in microvilli, which suggests an interaction with each other (66) (**Figure 3B**). They also proposed a role for ERM proteins, once phosphorylated (p-ERM), in connecting TCRs to the actin cytoskeleton of T lymphocyte microvilli (66) (**Figure 3B**). This interaction would hinder the dispersion of TCR molecules away from microvilli (66). Hence, preclustering of TCR prior to cognate pMHC binding occurs because of the prominent confined concentration of TCR on microvilli tips. This phenomenon has been proposed to heighten the sensitivity and selectivity of the T lymphocyte effector response by easing the interaction with numerous cognate pMHC molecules on the APC’s surface and allowing fast rebinding episodes (49). This strategy can thus increase the sensitivity of TCR for scarce cognate antigens upon TCR identification and contribute to the maintenance of the T lymphocyte activation responses.

### Polarized secretion of EV at the immune synapse

Advances over the last years have unveiled that cells exploit a myriad of distinct EV to ease communication both with neighboring and remote cells (68). A widely accepted view is that EV include a heterogeneous array of vesicles, with MVB-derived, classical exosomes representing only a minor fraction of these EV (68). It is also unclear whether further heterogeneity of EV might still be uncovered and which type of EV is responsible for each observed biological effect (69, 70). Remarkably, current isolation techniques for EV permit classification according to their surface antigen, density, and/or size, but do not distinguish EV based on their origin. This limitation prevents their classification as, for instance, ectosomes (EV produced by outward budding from plasma membrane) or exosomes (EV originated from intraluminal vesicles contained into MVB, see below), as these populations display overlapping size and composition (69, 70). Moreover, recent studies in breast cancer cells have shown the release of hypothetical “non-classical” exosomes generated by amphisomes (produced by fusion of multivesicular bodies -MVB- and autophagosomes) (71–73)), lacking classical exosome markers (CD63, CD81, and CD9). Recent excellent reviews have already dealt with EV generation, secretion, and heterogeneity in general (68, 70, 72, 73), please refer to these reviews since we will focus on EV secretion by T lymphocytes at the IS.

One major feature of EV secreted by T lymphocytes is the fact that their secretion is induced via TCR stimulation by antigen. Interestingly, plasma membrane receptor-induced EV secretion is restricted to certain cells from the immune system, including T and B lymphocytes upon TCR and BCR stimulation at the IS, respectively (48). TCR engagement by cognate pMHC and subsequent signaling at the IS leads to the establishment of a mature IS structured in the mentioned SMACs. This specific cortical organization of T cells relies on the redistribution of the actin cytoskeleton, which is an early and pivotal event following TCR stimulation (12, 29). Subsequently, the centrosome translocates towards the IS along with MVB and CG (28, 74), and then intraluminal vesicles (ILV) from MVB are released as classical exosomes (57, 58), and CG undergo degranulation, releasing perforin and granzyme to the synaptic cleft (11). However, although polarization is one of the main downstream consequences of IS signaling, the precise pathways and mechanisms governing MVB polarization and exosome secretion are still poorly understood.

TCR engagement by pMHC triggers the phosphorylation of ITAM, located in the cytosolic region of CD3 chains, by Lck. Then, TCR signaling [reviewed at (23, 28)] leads to the generation of a gradient of the early second messenger diacylglycerol (DAG) at cSMAC. DAG drives T cell polarization (14, 58) and regulates several protein kinase C (PKC) isoforms, Ras/ERK2 pathway, and protein kinase D (75). This leads to the stimulation of the two main actin-controlling pathways (formins and Cdc42/WASP/Arp2/3) (24, 76), while the remodeling of microtubule cytoskeleton is also induced (14, 18).

**Figure 4:**
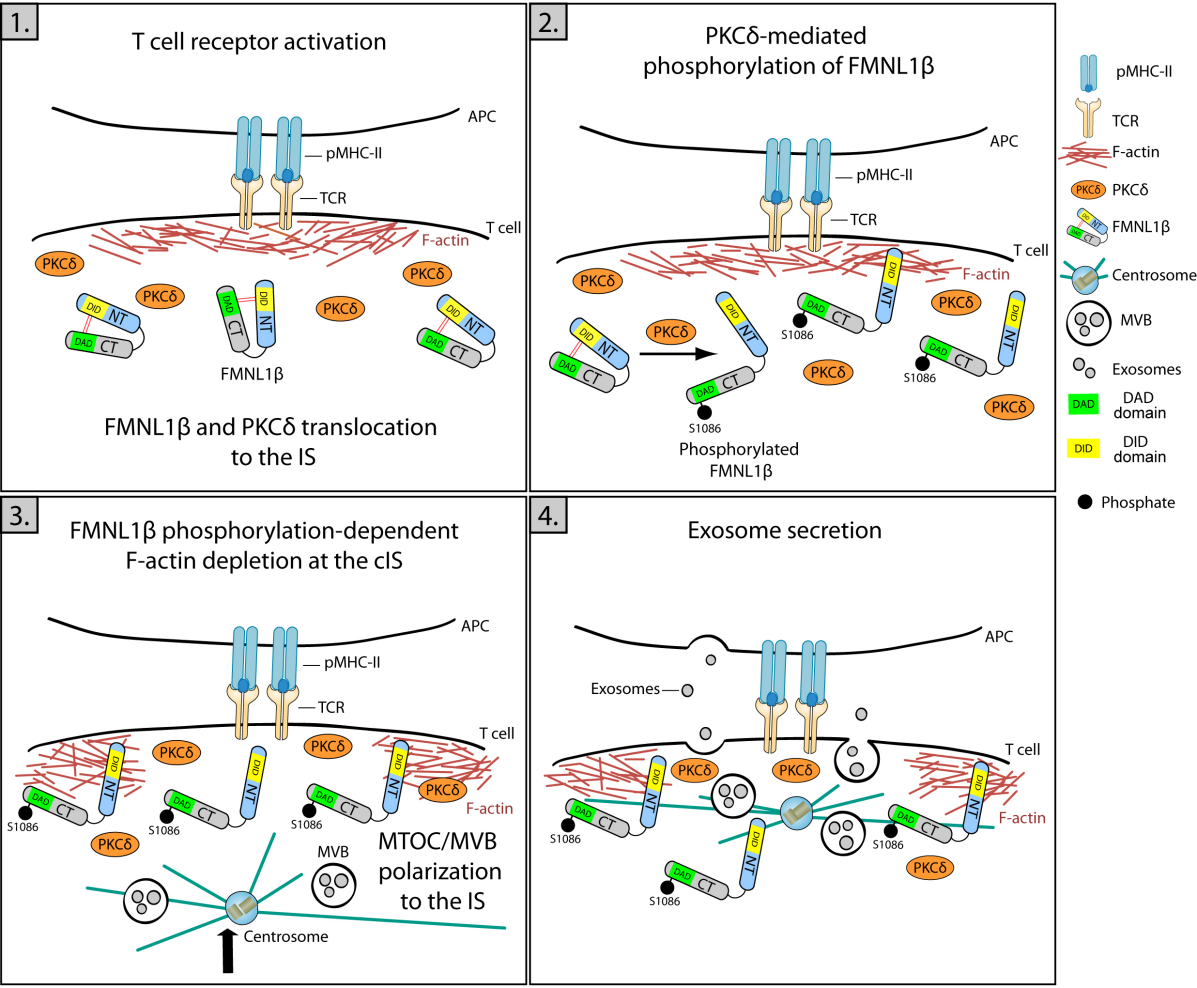
Role of FMNL1β in secretory polarized traffic and EV release at the IS. Schematic diagram depicting different steps in the polarization of MVB and exosome secretion in the Jurkat/SEE-Raji IS model. 1) IS formation is marked by an initial increase in cortical actin at the IS, where FMNL1β translocates and accumulates through a PKCδ-independent process. 2) FMNL1β undergoes a PKCδ-mediated phosphorylation at S1086, which reverses FMNL1β autoinhibition mediated by interaction of N-terminal Diaphanous inhibitory domain (DID) (yellow) with the C-terminal Diaphanous autoinhibitory domain (DAD) (green) and this may activate this formin at the IS. 3) Once phosphorylated at S1086, FMNL1β appears to govern the cortical actin reorganization process, creating a hypodense region at the cIS and the subsequent centrosome/MVB polarization to the IS. 4) These coordinated processes are crucial to enable a functional exosome release by T lymphocytes at the synaptic cleft (4, 18, 79).

Protein kinase C δ (PKCδ) activated by TCR triggering induces the reorganization of cortical actin (**Figure 1**; **Supplementary Video 2**) and the polarization of both the centrosome and MVB towards the IS (4), being also required for the CG polarization to the IS and subsequent cytotoxicity of target cells induced by mouse CTL (77, 78). Formins are implicated in F-actin nucleation and, among them, formin-like 1 β (FMNL1β) is recruited and transiently accumulated at the IS in Jurkat T lymphocytes in a process independent of PKCδ (79). Once at the IS, FMNL1 and other formins such as Diaphanous-1 (mDia1), are augmented at the heads of F-actomyosin spikes at the dSMAC and induce the generation of actomyosin arcs at the pSMAC (42). Moreover, the FMNL1β isoform at the IS undergoes a PKCδ-dependent phosphorylation (18) at the S1086 residue located at C-terminus (79). Remarkably FMNL2, a closely related formin that displays an elevated homology with FMNL1β, undergoes phosphorylation at S1072 by PKCα and, to a lesser degree, by PKCδ (80). S1072 phosphorylation reverts the FMNL2 autoinhibition controlled by N-terminal Diaphanous inhibitory domain (DID) binding with the C-terminal Diaphanous autoinhibitory domain (DAD). The reversed autoinhibition results in enhanced F-actin assemblage, β1-integrin endocytosis, cell invasive movement (80), and filopodia elongation (81). Therefore, it has been hypothesized that, upon IS formation, PKCδ-mediated phosphorylation at S1086 residue, located in the DAD domain within the C-terminal region of FMNL1β, may liberate the DID/DAD-controlled autoinhibition. This triggers FMNL1β activation and F-actin restructuring at the IS and, consequently, mediates MVB/centrosome polarization and exosome release (18, 79) (**Figure 4**). This FMNL1β S1086 phosphorylation mediated by PKCδ has been demonstrated to be essential for the generation of a hypodense cortical actin area in the cIS and for the polarization of the centrosome/MVB towards the IS (4, 18). These processes, essential for the functional release of exosomes at the IS, establish FMNL1β, through its PKCδ-mediated phosphorylation at S1086, as a fundamental regulator of polarized secretion by T lymphocytes (18, 79) (**Figure 4**).

## Extracellular mediators at the immune synapse

In the past 20 years, apart from several soluble stimulatory cytokines released at the synaptic cleft, diverse supramolecular complexes that transfer biological information across the synaptic interface have been characterized (61). These synaptic complexes include non-vesicular supramolecular attack particles (SMAPs) (59), and EV such as classical exosomes (57, 58), ectosomes (82) (60, 61), and T cell microvilli particles (TMP) (62, 83). SMAPs and exosomes are both released upon MVB degranulation at the synaptic cleft (58, 59), whereas synaptic ectosomes are generated by direct budding from the T lymphocyte plasma membrane, whereas TMP can also be released through either direct budding or trogocytosed from microvilli by target cell across the synaptic cleft (62, 83) (**Figure 3**). All these types of EV have been recently termed as trans-synaptic vesicles (61). In general, ILVs formation into MVB is a lipid-driven process, and during MVB maturation, endosomal sorting complexes required for transport (ESCRT)-dependent, but also ESCRT-independent pathways, are involved (84–86). These ESCRT-independent pathways for ILV generation seem to be directed by some specific lipids such as lyso-bisphosphatidic acid (87), ceramides (88), DAG (46, 58, 89, 90, 91), and certain tetraspanins such as CD63 (92, 93). In T lymphocytes both exosomes and ectosomes are EV actively generated and released through ESCRT (60, 61), the action of certain lipids and lipid-metabolizing enzymes (57, 58, 90) or tetraspanins (61) [reviewed in (46, 48, 93, 94)]. However, it has been recently published in a study using different cell types that despite being enriched at EV membranes, neither CD9 nor CD63 tetraspanins are needed for the EV uptake and cargo-delivery process (95). Moreover, exosomes produced by T lymphocytes may carry apoptosis inducer molecules such as Fas ligand (FasL) (90, 96). However, it is conceivable that further heterogeneity of EV at the IS can be uncovered in the future, provided that new toolsets for EV purification and characterization may be available (70).

CTL release different extracellular mediators to eliminate target cells (59). Recent studies support that the cytotoxic arsenal of CTL is stored in distinct, complex lysosome-related organelles (LRO) that undergo exocytosis to the synaptic cleft upon TCR stimulation at the IS (55, 97). These organelles encompass three main classes of cytotoxic entities, namely soluble cytotoxic proteins (i.e. perforin, granzyme), the SMAPs that carry encapsulated perforin and granzyme, and MVB carrying the proapoptotic FasL into their ILV (55) (**Figure 3**). These cytotoxic entities define three different pathways for target cell killing with the first two being carried out by non-vesicular entities and the third by a vesicular one (55). SMAPs are 100 nm nanoparticles encircled by a thrombospondin 1 crust and containing a core of cytolytic proteins including granzyme B and perforin 1. A recent report shows that SMAPs are present in multicore granules (MCG) with MVB structure and denote another wave of directed cell killing after the instantaneous release of soluble cytolytic proteins exerted by the canonical CG with single core granule (SCG) structure (55, 56) (**Figure 3**). Morphologically the LRO can be distinguished into at least two organelles called MCG and SCG (55, 56). Although it is clear that there are at least two classes of LRO with different protein composition, one enriched in FasL and the other enriched in perforin/granzymes, it is not clear whether the three abovementioned cytotoxic entities are executed by two or more different MVB-related MCG structures, whether these cytotoxic entities share or not the same LRO or whether these entities have redundant roles in CTL activity (55, 56, 59, 97). Whereas SCG containing soluble perforin and granzyme B correspond to the canonical CG are probably involved in the first wave of cytotoxicity (56), the different MCG, by releasing SMAPs and exosomes, would gain relevance as a second wave of attack (56, 98) (**Figure 3**), although more research is needed to solve all these issues.

Some authors have discussed that budding ectosomes and TMP could refer to the same biological entity, since both EV derive from high-positive curvature regions of the plasma membrane (67) and require vacuolar protein-sorting-associated protein 4 (Vps4) activity (60, 62, 83). Moreover, both EV appear to undergo trogocytosis (60), although this possible identity remains still unclear (67, 83). Since recent reviews have extensively dealt with exosomes and ectosomes at the IS (46, 48, 55, 97), we will focus on the recent work on EV derived from microvilli.

## Microvilli composition and function

Microvilli are actin-rich structures present on T lymphocyte surfaces where different receptors and signaling molecules cluster, thus enabling effective signal processing. Recently, several studies have focused on the different molecules, apart from TCR, that are present or exhibit differential localizations at the T cell microvilli tips (65, 67). There is evidence indicating the presence of CD2, the co-stimulatory adhesion molecule that binds to LFA-3, on microvilli (66). Also in this study, two signaling proteins positively implicated in the early TCR signaling events, LAT and Lck, were also discovered to be enriched on T cells microvilli tips, as TCR does. One study using a combination of super-resolution microscopy techniques exposed that CD45, a tyrosine phosphatase that negatively regulates TCR signaling, was found on T cell microvilli tips (65). However, a subsequent study of the same group demonstrated in T lymphocytes that CD45 is excluded from the tips of microvilli preceding APC engagement (27). Therefore, the spatial orchestration of these molecules leading to their specific preclustering at microvilli appears to facilitate the initial antigen recognition events and thus would sensitize and reinforce TCR activation signals (66). In this context, actin cytoskeleton together with ERM proteins acting as switchable phosphorylation-regulated linkers, appear to participate in the coordinated reorganization of different molecules, such as TCR, along microvilli (66).

In addition, lipids appear to play a crucial role in the generation of curved domains at the plasma membrane and microvilli formation. A recent review has already addressed this issue (99), and we recommend referring to this excellent report. Thus, both lipidic and proteic membrane heterogeneity indeed aid T cell activation, as previously discussed (49).

As stated in the Introduction, it is thought that T lymphocytes utilize the protrusions enriched in TCR at their tips to rapidly scan and search for cognate antigen bound to MHC molecules in APC (49). Such a topographic behavior may be considered as a “palpation” of the APC’s surface by microvilli enriched with TCR at their tips, which underlies the action of pMHC recognition by TCR (3) and delineates a crucial feature in cell-to-cell recognition, that is the time pressure of pMHC to consolidate discriminatory contacts with the surface of an opposing effector cell (3). Regarding their dynamic, the seemingly stochastic movements of microvilli caused a very rapid increase in accumulative APC´s surface scanning coverage over time and approximately 98% of the APC´s exterior is visited by the interaction of at least one microvilli within one minute (3). As a reference, the half-life of T lymphocyte-APC interactions *in vivo* is approximately one minute, evoking that almost complete surveying of APC’s surface can be performed at physiological dwell times (3). Thus, these membrane ultrastructures on scanning T lymphocytes are especially dynamic in order to guarantee the compromise between antigen scanning velocity and sensitivity, as well as cognate antigen discrimination leading to antigen specificity.

Apart from all these activation-conferring features awarded by TCR and several relevant accessory molecules located on microvilli, it has been recently demonstrated that when microvillar interaction-size is enlarged by increasing the stabilization of CD2-ligand interactions on supported lipid bilayers (SLB), strong activation signals occur on T lymphocytes that do not depend on antigen. This suggests microvilli provide T lymphocytes with discriminatory receptor signaling capability conferred by the small contact sizes (100).

Last, but not least, the glycocalyx of APC and target cells are constituted of diverse bulky proteins and proteoglycans, that act as a physical barrier for TCR on microvilli tips. Microvilli protrusive tips may puncture through APC’s cell surface steric barriers and thereby actively locate the relatively smaller TCRs at high concentrations in close vicinity to pMHC presented by APC so that productive survey and subsequent interactions may efficiently occur (52). Thus, physical obstacles such as APC’s cell surface matrix and the glycocalyx are overwhelmed by the surveying and puncturing microvilli (52, 100).

In summary, it appears that given the time and spatial constraints for T lymphocytes to contact with APC (the duration of cell interaction is between 1-5 minutes) (101), they must be highly efficient in antigen detection and discrimination, but also in subsequent TCR-induced signaling. This would be guaranteed by the concurrence of several facts: 1, microvilli facilitate antigen survey by participating in serial and parallel short-life (few seconds) palpation (3) (**Supplementary Video 1**); 2, once cognate pMHC is recognized, microvilli containing TCRs at their tips are stabilized, increasing their half-life between 2-5 times compared to unengaged microvilli (3); 3, microvilli appear to ease TCR-pMHC interaction, as they move towards the APC within 15 nm (3, 101) (100), which is the length of the TCR-pMHC complex; 4, TCR accumulate in the narrow areas of close contact at the microvilli tips upon pMHC recognition (3, 53, 65); 5, microvilli, due to their thin structure, are highly fragile prone to be, sheared and easily detached as TCR-containing, T cell membrane particles (TMP, see below) by trogocytosis, producing a new group of EV (83) capable of signaling (see below); 6, relevant signaling molecules (CD45, Lck, LAT) redistribute to concentrate into or to be excluded from microvilli tips, thus fine-tuning activation signals. For all these reasons microvilli are considered as very effective and discriminatory sensors for cognate antigens. The palpation of the opposite APC surface by dynamic microvilli is the cause of both a rapid TCR recognition and discrimination of cognate pMHC, allowing activation signals amplification (3, 100, 102).

## Microvilli-derived extracellular vesicles

In comparison to the early description of EV from other sources, EV derived from microvilli have been recently characterized, and, as a consequence, much less is known regarding the molecular mechanisms involved in their biogenesis and their functions. The dynamic properties of microvilli together with their minor dimensions have impeded their functional and structural characterization for many years (67). While electron microscopy (EM) offers the highest spatial resolution in morphologic studies, understanding the dynamics and function of microvilli in signaling requires the characterization of their morphology and the localization of signaling proteins with high spatio-temporal resolution in living cells. The recent progress of super-resolution fluorescence microscopy techniques has allowed a myriad of studies of protein localization and membrane morphology in microvilli at high spatial and temporal resolutions, although the combination of both capabilities is still a technological challenge (3, 27, 53, 62, 65, 66, 100) (see below).

Several results have shown that microvilli constitute an exclusive compartment that senses and processes signals conducting to efficient T lymphocyte activation (see above). However, recently it has been established that microvilli are not only limited to antigen-sensing, they also act as effector entities. This is largely attributed to their ability to produce TMP, which in turn modulates crucial T lymphocyte effector functions such as cytotoxicity, cell activation, and clonal expansion (51, 53, 62–64, 83). While it is true that other EV exhibit important effector functions that can send molecular messages over a distance (48), in the case of microvilli trogocytosis, the produced EV are released via a strict cell-cell contact-dependent manner over short distances and require APC’s active contribution (83). However, it is noteworthy that the interfacial F-actin protrusions mechanically increase target cell death by CTL. Perforin is secreted at the bottom of these F-actin-rich protrusions (51), although no participation of TMP from these microvilli in target cell killing was established in the last report. Taken together, microvilli participate in several effector functions of T lymphocytes, including cytotoxicity (51), and regulate cell activation/proliferation of both APC (62) and T lymphocytes (53, 63).

To study the mechanisms of TMP release, it is necessary to differentiate between those that are trogocytosed by an APC and those that, conversely, are directly liberated from the microvilli membrane. Regarding direct vesicle budding, Arrestin-domain-containing protein-1 (Arrdc1) is exclusively localized in the plasma membrane and mediates both the formation and liberation of microvesicles directly from there (62). This process can be achieved by using the enzyme vacuolar Vps4, which has ATPase activity, and the tumor susceptibility gene 101 (TSG101) belonging to the ESCRT (62, 103, 104). In agreement with this result, an article demonstrated that TSG101 organizes TCRs at the center of the IS and, subsequently, Vps4 is responsible for mediating the excision of TCR-enriched membrane vesicles from the membrane (60). One interesting study revealed that Arrdc1 is highly concentrated at microvilli tips, colocalizing with TCR, and relocated toward the cSMAC through the IS evolution process (62). TSG101 was also found at microvilli when co-expressed with Arrdc1 (62). This study also established that these proteins, together with Vps4, create membrane budding complexes that trigger the direct release of vesicles from the microvilli membrane.

Regarding the trogocytosed vesicles, the detailed mechanism by which this process occurs is still unknown. It has been hypothesized that fragments of the cell membrane may be extracted by the tensile force produced by interacting cells (62, 83). Additionally, the elevated avidity of receptor interactions has also been proposed as a potential cause for the trogocytosis process (5, 83). Finally, the selective microvilli enrichment not only with TCRs or adhesion molecules, but also with membrane sorting complexes such as Arrdc1, TSG101, and Vps4 (involved in direct vesicle budding) suggests that the generation of TMP through trogocytosis requires the action of enzymatic complexes in the effector T lymphocyte. This means that although the T lymphocyte interaction with the APC is needed for TMP to be produced, it is not a passive or an exclusively APC-mediated process (83), and a collaboration between effector T lymphocytes and APC is needed.

In relation to microvilli dynamics and TMP production in the context of IS formation and resolution, it is remarkable that several studies in living, effector CD8^+^ T lymphocytes indicated that microvilli quickly move laterally and flutter, and this allows a rapid and efficient survey of APC´s surface for cognate pMHC (3). At the early stage of IS creation, numerous TCR and signaling molecules-enriched microvilli are polarized onto the APC’s surface containing cognate pMHC for recognition (64, 83). The microvilli dynamics slows down once the contact and adhesion with the APC stabilizes as a consequence of the productive encounter of cognate pMHC by TCR and TCR-induced inside-out activation of integrins such as LFA-1 and VLA-4 at the IS (31, 64). At the mature, second stage, when T lymphocytes are fully activated, the initially elongated microvilli almost disappear, although some remain at the outermost border of the T-activated T lymphocyte (64). The combination of the tight synaptic contact and the long microvilli initially enhances cell adhesion and spreading of the T lymphocyte on APC, and subsequently promotes contact-dependent transmission of large microvilli fragments to APC. During the third terminal, or IS contraction stage, T lymphocytes shed abundant TMP on the APC’s surface. Moreover, substantial surface-anchored, large TMP undergo further fragmentation into nano-sized TPM, through the involvement of APC’s plasma membrane budding complexes, which include Arrdc1, TSG101, and Vps4 (64, 83) (**Figure 3**).

In addition, as stated by some authors (64), it is intriguing that T lymphocytes appear to use the same organelle (microvilli) to sense cognate antigens and to produce effector EV. It is remarkable that this situation does not occur in other organelles (i.e. MVBs) involved in the secretion of different EV, such as exosomes. However, from our point of view, this double function exerted by this organelle may underpin the mechanism by which TCR discriminates quality and quantity of pMHC, and T lymphocytes transmit feedback to APCs, which has been suggested to be proportional to the amount of antigen presented in cognate pMHC presenting cell (105). This double function of T lymphocyte microvilli may also participate in Th lymphocyte-DC and/or CTL-target cell interactions. It has been suggested that the T lymphocyte “antigen counting” capability, exerted via TCR-containing shedding vesicles, would program B lymphocytes to respond proportionally to the number of pMHC they present to Th cells (105). The reception by B cells of a limited number of TCR-enriched ectovesicles from T lymphocytes has been proposed to support activation/division of the B cell until lost by dilution, thus endowing the B cell with an “antigen counter” capability for antigen (105). Thus, TCR-enriched-TMP may also participate in these mechanisms. Moreover, microvilli sense and discriminate the cognate antigen and may also produce the proportional and accurate quantity of effector TMP. This sophisticated, bidirectional double-counting antigen mechanism may provide T lymphocytes with an essential function for antigen response using the same organelle, thus saving important or limiting biological resources. Future experiments will establish whether these two functions of microvilli (antigen sensing and TMP production) can be segregated and will study the relative contribution of each of these functions to T lymphocyte and APC activation (see below).

In this context of TMP possible functions, results from different groups support that ectocytosis through microvilli removes activated TCRs from the membrane of CTL (53, 63), terminating signaling as ectosomes are taken up by dying target cells, and allowing CTL and target cell to disengage where ectosomes bud off from the CTL membrane, facilitating subsequent serial killing and self-limiting TCR signaling (53). In one of these reports, the initial loss of membrane TCR by shedding, involving both trogocytosis and enzymatic vesiculation processes, surprisingly results in rapid TCR upregulation, metabolic reprogramming, and enhanced cell division and survival (63). Thus, TMP secretion results in profound changes in TCR signaling and T lymphocyte effector functions. For other authors, TMP from effector CD4^+^ lymphocytes enriched in diverse adhesion molecules and several cytokines are capable of activating DC independently of cognate TCR-pMHC signaling and thus has been considered as bona fide “immunological synaptosomes”, collaborating in APC activation (62, 64). In these experiments, TCR and several T lymphocyte surface proteins enriched in microvilli such as CD4, CD2, CD28, and CD25 were transferred to the DC’s surface, together several cytokines contained into TMP, such as IL-4, IL-7, TNF-α and IL-33, contributing to DC activation (64). Thus, TMP are enriched with relevant T lymphocyte signaling proteins and well-established regulators of DC activation and differentiation, and indeed may contribute to immune cell communication. In general, it may be concluded that T lymphocytes are highly mobile cells specifically designed to circulate throughout the entire organism, actively surveying for cognate antigens and delivering messages to a wide variety of cells. Hence, it can be speculated that T lymphocytes have evolved to hostage specialized strategies to promote cell communication with interacting cells, even within a mobility context (62). In this sense, both microvilli-based scanning and message transfer via TMP might represent the unique manner of sensing antigen and provide communication with other immune cells or non-mobile cells resident in distinct tissues through the same cell structure (62, 64). However, further research is essential to establish the mechanism involved in TMP internalization and their role in receptor cell activation. Noteworthy, TMPs are proposed to participate only in communication via physical interaction of the microvilli with the surface of cognate APCs, in contrast with exosomes and ectovesicles which are also proposed to participate in long-distance communication (83).

## Current research gaps

Results from different laboratories sustain that different types of EV can be produced at the T lymphocyte side of both the Th and CTL IS (48) (**Figure 3**). In this context, please consider that the International Society of Extracellular Vesicles (ISEV, https://www.isev.org/) endorses “extracellular vesicle as the generic term for particles naturally released from the cell that are delimited by a lipid bilayer and cannot replicate, i.e., do not contain a functional nucleus” (69). This comprises classical exosomes (endosomal origin), non-classical exosomes (amphisome origin), microvesicles, ectosomes (different classes of plasma membrane-shedding vesicles), TMPs (**Figure 3**), and even apoptotic bodies. The “minimal experimental requirements for definition of extracellular vesicles” from ISEV specify endorsements on “methodology and minimal information on reporting EV isolation/purification, EV characterization, and studies on EV biological function” (106). Since exosome composition and biogenesis are out of the scope of this review, please consult several relevant reviews on this subject (68, 93, 94, 107, 108), in particular those recent reviews dedicated to EV from T and B lymphocytes (46, 48, 72). It is clear from these reviews and ISEV statements that there are not “exosome-specific markers or EV-specific markers, but rather exosome-enriched (or EV-enriched) proteins”. In this context, CD81 and CD63, which are augmented in classical exosomes secreted by a myriad of cell classes, have been proposed as classical exosome markers (94). However, they also appear at the plasma membrane of T lymphocytes, most likely as a result of constitutive, multidirectional MVB degranulation (58, 109) (**Supplementary Video 1**; **Figure 1**). Consequently, CD63 and CD81 can be found in shedding vesicles, microvilli (**Supplementary Video 1**; **Figure 1**), TMPs and apoptotic bodies, as all of these are plasma membrane-containing EV. In this context, ISEV has endorsed a record of EV-specific markers (69, 106), but has not succeeded to distinguish among EV subclasses (69, 106, 110).

Recently, a report demonstrated that cells use a shared, stochastic procedure to sort exosome cargoes along the range of endosome and plasma membranes, which is far more efficient from the plasma membrane than from the endosome (111). This leads to a significant content variation among distinct exosome-sized vesicles, which is an inexorable outcome of the stochastic mechanism governing small vesicle generation, thus the source membrane of exosome-sized EV basically cannot be established (111). Specifically, the consequence of these single-exosome analyses (111) is in conflict with the notion that there are different subclasses of exosome-sized vesicles or that exosomes can be distinguished from ectosomes based, for instance, on CD9/CD63 proportions, as suggested by other authors (112). In summary, unless definitive proof of their biological origin (MVB, amphisomes, plasma membrane, microvilli, dying cell) is obtained by imaging or alternative techniques, the generic term EV should be used for these extracellular entities. Further dissection of EV heterogeneity and phenotyping (68), needs the development of high-throughput, multiparameter analysis methods (112) of single vesicles (113) combined with high spatial resolution imaging (114). In this context, it is remarkable that improved methodology for EV isolation and characterization has revealed the existence of “non-classical” exosomes (CD63-, CD81-, CD9- negative exosomes) produced from amphisomes in a breast cancer cell line (71), and thus this new EV type may also be present in the IS. There is a general consensus among researchers that the outcome of EV studies greatly depends on the methodology used for EV isolation and characterization, since minor alterations in protocols can direct to major alterations in outcomes and deductions (68, 70, 73), since these protocols are not specific for a particular EV class. The heterogeneity and low levels of material in EV preparations require new sensitive nanoscale methods and isolation approaches. This represents a particular challenge and likely explains why a standardized toolset to properly study EV has not been established yet (70).

Accumulated evidence shows that both actin cytoskeleton remodeling and polarized secretory traffic occur not only at the T lymphocyte effector side, but also at the APC or target cell side of the IS (98, 115). Although it is out of the scope of this review to deal with this subject due to space limitations, there are several parallelisms between the events occurring on both sides of the IS that could contribute to guide future research. For example, the tumor side of the CTL synapse has been much less studied to date (115). The cytoskeleton changes and secretory traffic occurring at the tumor side indeed contribute to modulating the T lymphocyte immune response at the IS, and ultimately may facilitate the escape of tumor cells to the immune control (115, 116). These events include cortical actin cytoskeleton reorganization (115) and may involve polarized traffic of MVBs and exosome secretion [among other polarized secretion events reviewed in (116)] at the synaptic cleft from the tumor side (117). Based on the fact that tumor cells can recruit and polarize their actin cytoskeleton and secretory lysosomal compartment to the IS, neutralizing CTL cytotoxicity (116, 118), it is conceivable that they also have the capability to polarize MVB and to focus immunoregulatory exosome secretion to the IS (117). However, a formal relationship between actin cytoskeleton and polarized MVB secretory traffic such as the existing in T lymphocytes or NK cells, both in effector Th or cytolytic synapses, has not been formally demonstrated yet to occur in tumor cells forming IS. Otherwise, this would render tumor cells as polarization-competent, exosome secretion effector cells. In addition, the microvilli also emanate from the target tumor cell or APC side in the IS (**Figure 1**) and it is unknown whether these microvilli may contribute to the synaptic TMP secretion by the tumor cell. More research is necessary to establish these important points, since this indeed will construct a global and systemic view on the role of EV at the IS, and will increase our understanding of some of the processes regulating the escape of tumor cells to the immune control.

The application to IS studies of optic super-resolution microscopy techniques possessing high spatial and temporal resolution, for instance, lattice light sheet microscopy (LLSM) (119), applied to IS studies (3, 29), has brought new light to the contribution of microvilli to antigen detection by T lymphocytes. For adequate live-cell imaging using fluorescence microscopy, both for IS in general and for microvilli in particular, it is essential to strike a balance between spatial and temporal resolutions, to overcome the spatial restrictions produced by imaging in Z optical axis, to improve signal to noise ratio, and to solve cytotoxicity and sample photobleaching intrinsic to any live cell imaging method (37, 120, 121). Frequently, challenges in IS visualization at high spatiotemporal resolution have somewhat limited our knowledge of the synaptic architecture and the molecular bases that drive T lymphocyte activation (38). Recently, visualization and quantification of IS dynamics have benefited from advances in spatiotemporal resolution of microscopy techniques (38). At this moment, our understanding of how IS architecture controls synaptic signaling and patterning is limited. This is due to various facts combining the rapid kinetic variations, the compact region of cell-to-cell synaptic contacts, and the highly malleable and uneven cell-cell synaptic interface that impedes image acquisition at high spatial and temporal resolution (32, 38). However, the progress of emergent techniques such as LLSM (3, 21) and 3D live-cell super-resolution microscopy (122), is poised to revolutionize our approach to image protein and cellular dynamics in the course of synaptic interactions (54). These progresses are certain to improve our understanding of these intricate processes.

In the meantime, several experimental approaches have been employed to overcome the abovementioned limitations. Planar SLB or beads coated with agonistic antibodies or cell-surface proteins acting as surrogate antigens are preferred options (see below). These approaches reduce the intricate 3D structure of cell-to-cell IS to two dimensions, facilitating the use of high-resolution imaging techniques including total internal reflection fluorescence microscopy (TIRFM) (7) and, because cell stimulation occurs at an evenly and well-identified Z position, image capturing with higher spatial resolution becomes viable (65, 123). If the movement in the Z dimension is confined or the imaged cell is flat enough, secretory vesicle movement, microvilli, or F-actin reorganization at the XY focus plane can be conveniently imaged and analyzed in living cells (3, 8, 65, 101, 124). Under this technique, the images acquired by using artificial lipid bilayers and anti-TCR-coated coverslips, in combination with TIRFM and total internal reflection fluorescence–structured-illumination microscopy (TIRFM-SIM) (42, 101, 124) or 3D single-molecule localization microscopy (3D-SMLM) (125), probably exhibit the highest spatial resolution and definition achieved until now.

Regarding the possibility of functional and structural heterogeneity not only in EV but also in microvilli or other F-actin-rich protrusions, there is evidence supporting that the functionality of synaptic protrusions in CTL is partitioned both spatially (between protrusions at the IS center versus at the IS periphery) and molecularly (participation of either WASP or WAVE2) (51). These findings may explain the early results showing WASP is not involved in microvilli morphogenesis (50), in apparent contradiction with a recent report (51). Thus, more research is needed to establish this important point since this issue may indeed have strong implications for microvilli and TMP generation and function.

It has been demonstrated that the distribution of microvilli at the IS interface undergoes reorganization over time (62, 83). In this study, it is observed that during the early stages of IS formation, microvilli undergo polarization towards the surface of the APC. Furthermore, they show that once the synapse matures, the number of microvilli is reduced, and the remaining ones are organized in the distal areas of the IS, in the dSMAC. This resembles the positive and negative changes in F-actin reorganization that occur during IS maturation, which are crucial for both EV and cytokine polarized release (20, 21, 28), but also constrain EV secretion at the late stage of the IS (30). The causes governing this microvilli reorganization, leading to an initial increase in their density at the contact synaptic zone and subsequently, to a reduction in the cIS, remain unknown. Considering that microvilli are F-actin-rich structures, it seems feasible that F-actin depolymerization in the cIS, which facilitates the polarized secretory function (20, 21, 28), may affect negatively the polymerization or stability of microvilli in that specific region. Alternatively, both microvilli and F-actin may gradually be eliminated from the cSMAC as a consequence of trogocytosis by the APC.

Finally, since knockdown of either Vps4 or Arrdc1 revealed minor effects on microvilli structure but TMP generation was reduced (62), it will be interesting to test in the synapses developed by T lymphocytes lacking Arrdc1 or Vps4, both the antigen scanning capability leading to T lymphocyte activation and APC activation. Results from these experiments may help to establish whether the two functions of microvilli (antigen sensing and TMP production) can segregate and to study the relative contribution of each microvilli function to T lymphocyte and APC activations.

In addition, further experiments involving more “selective” protein markers are required to clarify whether budding ectosomes and TMP share the same biological origin, which remains a controversial issue (67, 83).

## Future directions and concluding remarks

**S**ome imaging considerations related to IS studies in general, and EV studies within the IS in particular, may be pose difficulties or even fully limit the interpretation of EV results. The majority of the state-of-the-art imaging and investigation of IS architecture and F-actin dynamics is founded on artificial planar synapses achieved using APC surrogates, by either embedding stimulatory and co-stimulatory molecules into lipid bilayers or by coating these molecules on plastic or glass surfaces (12, 34, 37, 42, 126, 127). However, these planar synapses do not reflect the 3D structure of real IS.

In this context, most of the recent synaptic EV imaging studies at the synapse have been performed using second-generation beads supported lipid bilayers (BSLB) covered with a surrogate antigen, which allows EV elution and thus facilitate EV characterization analyses (61), or using flat SLB (3). Indeed, both SLB types are superb means to analyze the biogenesis of both synaptic EV and SMAPs, since trans-synaptic particles are rapidly internalized by “real” APC (61) but not by BSLB. In addition, BSLB surfaces can be designed, defined, and reconstituted with different concentrations of surrogate antigens and diverse adhesion molecules (100), as well as lipids (7), allowing dissection and purification/reconstitution approaches (10, 61, 126). In addition, since BLSB lack trogocytic capabilities, they may contribute to dissecting the contribution of trogocytosis and/or actin reorganization to microvilli regression at the cIS when IS matures. Thus, to study processes in which APC play an active role in EV generation (i.e. trogocytosis favoring TMP production) (83), the most appropriate, least reductionist, cell-cell synapse models must be used, mainly when high 3D spatiotemporal resolution is required. Some cell-cell synapse models (40, 57, 58, 109) mimic the complicated and uneven interactions existing on the 3D stimulatory surface of a physiological IS better than artificial synapses do (12, 18, 126). Moreover, it has been already discussed how adherence to some stimulatory artificial surfaces can unintentionally activate T lymphocytes and remains a polemic discovery that illustrates the complexity of imaging depending on stimulatory surface class, preparation, and composition (49, 54). Moreover, the use of a stiff activator surface may mechanically obstruct cells and impede basic membrane formations (49). Taken together, the use of several imaging techniques using artificial APC substitutes has unquestionably been an advantage to understanding how these intricate immune cells operate, nevertheless, these approaches are intrinsically limiting (49, 54). Then, it should be considered how these findings may relate to complex 3D interactions between an APC and a T lymphocyte (49). Thus, studies with SLB and BSLB are compelling in terms of spatiotemporal resolution and sensitivity, although it is imperative to check the predictions of these models in *in vitro* cell-to-cell or *in vivo* approaches (126). In this regard, approaches based on agonist-coated stimulatory surfaces in combination with cell-cell synapse models may provide complementary and useful information (62).

Regarding emerging imaging techniques applied to microvilli studies, high-resolution LLSM and TIRFM have been recently used to study T cell microvilli interactions exerted by chimeric antigen receptor (CAR) T lymphocytes used for cancer treatment (128), which was performed in developing synapses (129). In this report, CARs were found in microvillar contacts distributed similarly to TCRs, but higher CAR affinity compared to TCR affinity led to microvilli hyperstabilization, anomalous IS resolution, diminished effector function, and enhanced tendency to CAR T lymphocyte exhaustion, which could be relieved by decreasing CAR avidity or affinity (129). Although in the last report the contribution of TMP to the observed microvilli-controlled effects was not assessed, emerging imaging techniques such as LLSM and 3D live-cell super-resolution microscopy will indeed improve our understanding of microvilli function and will also allow us to analyze microvilli structure and function and their derived TMPs in the framework of modifications in CAR design (130). Indeed, these advances will have profound implications for CAR-based cancer immunotherapy.

In addition, microvilli have been proposed to function as immunological “synaptosomes”, carrying TCR and competent signaling molecules, enabling interactions with APC and promoting the transmission of antigen-specific activation messages to APC (62). Thus, these super-resolution techniques will shed more light on the ultra-structure of the microvilli tips at the synaptosome-APC contact area, and this will allow us to establish whether this area resembles or not, at the nanoscale level, the SMAC-based structure of the canonical IS.

The fact that T cell activation molecules (TCR, CD4 coreceptor, CD2 adhesion molecule, and signaling molecules such LAT and Lck) have been found enriched in microvilli tips (52, 66), whereas CD45 and LFA-1 are excluded from microvilli (3, 27), support that a selective sorting machinery for partitioning these molecules on microvilli must exist. Whereas it is accepted that the pre-arrangement of the TCR and its signaling machinery on microvilli rests on their anchorage to actin cytoskeleton assisted by activated ERM linkers (66), what drives the general organization of signaling molecules in and out the microvilli remains elusive (52). However, it is speculated that the lipid composition and the extreme membrane curvature may play and important role (49, 67). In this line, cholesterol content plays a role, since CD45 is excluded from the microvilli tips in a manner contingent on cholesterol-dependence (27). Since the distribution and segregation of T cell activation molecules on microvilli appears to be important for antigen recognition and activation, but also for EV production (52, 67, 83), further investigation is needed to establish the nature of the mechanisms implicated in these sorting and partitioning processes. Due to the existing resemblances between primary cilia and the IS exemplified by the participation of intraflagellar transport proteins in the effective sorting and transportation of several proteins to the ciliary membrane, but also in LAT recruitment to the IS membrane (131), it is tempting to analyze in the future the role of several well-known ciliary molecular effectors in protein sorting to the microvilli membrane.

In summary, some elegant, recent examples of using BSLB in combination with cell-cell synapse models are indeed preferred approaches that have profoundly changed our view of synaptic architecture and the contribution of microvilli and EV derived from microvilli to IS function (62, 63, 100). The development and incorporation of high-temporal resolution, super-resolution imaging techniques (28, 37, 54, 65, 70) to synapse studies, such as those recently published in CAR synapses (129, 132), will indeed improve our knowledge of the IS structure and function.

## Author contributions

JR-N: Formal analysis, Investigation, Writing – original draft, Writing – review & editing, Conceptualization, Methodology, Visualization. VC: Formal analysis, Investigation, Visualization, Writing – original draft, Writing – review & editing, Validation. MI: Conceptualization, Data curation, Formal analysis, Funding acquisition, Investigation, Project administration, Resources, Supervision, Validation, Visualization, Writing – original draft, Writing – review & editing, Methodology.

## Supplementary material

Supplementary Video 1 CMAC-labelled Raji cells (blue) were attached to ibiTreat 8- microwell culture dishes (ibidi) pretreated with fibronectin (0.1 mg/ml) and were pulsed with SEE superantigen for 30 min, to induce IS formation. Subsequently the Jurkat cells, previously transfected with GFP-CD63, were co-cultured with the SEE-pulsed Raji cells, thus all the plasma membrane protrusions (microvilli) decorated with GFP-CD63 belong to the Jurkat cells. GFP-CD63 is located at the limiting membrane of MVB (GFP-CD63^+^ vesicles) and at the plama membrane of Jurkat cells as a result of constitutive, multidirectional MVB fusion to the plasma membrane and subsequent CD63 relocalization via diffusion (58). Double IS formation by one GFP-CD63-expressing Jurkat cell (center) was time-lapse imaged (10 fps). Raji cells act as APC, whereas Jurkat cell is a model for Th lymphocyte. The video shows the Jurtat cell at the center developing two IS and the polarized traffic of MVB (GFP-CD63^+^ intracellular vesicles) towards the contact areas in the Jurkat cell forming the IS. This phenomenon is not observed in the other two Jurkat cells that do not form IS (left). White arrows label some microvilli projections belonging to the Jurkat cell labelled with GFP-CD63, which appear to actively palpate the APCs. The original video was published in Mazzeo, C., Calvo, V., Alonso, R. et al. “Protein kinase D1/2 is involved in the maturation of multivesicular bodies and secretion of exosomes in T and B lymphocytes”. *Cell Death Differ* **23**, 99–109 (2016). https://doi.org/10.1038/cdd.2015.72., please refer to this publication for additional details, and is shown here reprinted with permission from this reference, as a courtesy from SpringerNature (License Number 5624151303437).

Supplementary Video 2 C3 control (left) and P5 PKCδ-interfered (right) Jurkat clones were challenged with CMAC-labelled (blue), SEE-pulsed Raji cells to induce IS formation, as indicated in the **Figure 1** legend. After 1 h of conjugate formation, fixed cells were labelled with phalloidin (green), anti-CD63 (magenta) and analyzed by confocal fluorescence microscopy using the 3D Viewer plugin of the ImageJ software, to visualize Z planes and to project the IS interface (133). Upper panels: MIP of the merged CMAC, anti-CD63 and phalloidin channels. Lower panels: enlarged XZ-plane area of the IS interface (1.5x and 2.5x zoom for C3 and P5, respectively, from the white rectangles in first frames) of phalloidin and anti-CD63 channels. White arrows indicate the direction to visualize the IS interface views enclosed by the ROIs (white rectangles). Frame no. 1 of the video (7 fps) corresponds to the top view shown in **Figure 1** and, after a 3D rotation, last frame corresponds to the IS interface view shown in **Figure 1** , lower panel. The depletion of F-actin at the synaptic central region, is observed in the C3 control clone, but not in the P5 PKCδ-interfered clone. This video is related to **Figure 1**. See also (4, 133).

## Glossary

**Table:**

AICD	activation-induced cell death
ADAP	adhesion and degranulation adaptor protein
APC	antigen-presenting cell
Arrdc1	arrestin-domain-containing protein-1
BSLB	bead-supported lipid bilayer
CAR	chimeric antigen receptor
CMAC	cell tracker blue-CellTracker™ (7-amino-4-chloromethylcoumarin)
cIS	central region of the immune synapse
cSMAC	central supramolecular activation cluster
CG	cytolytic granules
CTL	cytotoxic T lymphocytes
DAD	diaphanous autoinhibitory domain
DC	dendritic cells
Dia1	Diaphanous-1
DID	diaphanous inhibitory domain
dSMAC	distal supramolecular activation cluster
EM	electron microscopy
ERM	ezrin, radixin, moesin
ESCRT	endosomal sorting complex required for transport
EV	extracellular vesicles
F-actin	filamentous actin
Fact-low cIS	F-actin-low region at the center of the immune synapse
FasL	Fas ligand
FMNL1	formin-like 1
fps	frames per second
ICAM-1	intercellular adhesion molecule 1
ILV	intraluminal vesicles
IS	Immune synapse
ITAM	immunoreceptor tyrosine-based activation motifs
LAT	linker for activation of T cells
Lck	lymphocyte-specific protein tyrosine kinase
LFA-1	lymphocyte function-associated antigen-1
LLSM	lattice light sheet microscopy
LRO	lysosome related organelles
MCG	multicore granules
MHC	major histocompatibility complex
pMHC	antigenic peptide/major histocompatibility complex
MIP	maximal intensity projection
MVB	multivesicular bodies
PKC	protein kinase C
PKCδ	protein kinase C δ
pSMAC	peripheral supramolecular activation cluster
ROIs	regions of interest
SCG	single core granules
3D-SMLM	three-dimensional single molecule localization microscopy
SEE	*Staphylococcal* enterotoxin E
SLB	supported lipid bilayer
SMAC	supramolecular activation cluster
SMAPs	supramolecular attack particles
TCR	T-cell receptor for antigen
Th	T helper
TIRFM	total internal reflection fluorescence microscopy
TIRF-SIM	total internal reflection fluorescence–structured-illumination microscopy
TMP	T cell microvilli particles
TSG101	tumor susceptibility gene 101
Vps4	vacuolar protein-sorting-associated protein 4
